# The effect of bacteriophages T4 and HAP1 on *in vitro *melanoma migration

**DOI:** 10.1186/1471-2180-9-13

**Published:** 2009-01-20

**Authors:** Krystyna Dąbrowska, Grzegorz Skaradziński, Paulina Jończyk, Aneta Kurzępa, Joanna Wietrzyk, Barbara Owczarek, Maciej Żaczek, Kinga Świtała-Jeleń, Janusz Boratyński, Gryzelda Poźniak, Magdalena Maciejewska, Andrzej Górski

**Affiliations:** 1 Institute of Immunology and Experimental Therapy, Polish Academy of Sciences, ul. R. Weigla 12, 53-114 Wroclaw, Poland; 2 Wroclaw University of Environmental and Life Sciences, Faculty of Veterinary Medicine, C.K. Norwida 31, 50-375 Wroclaw, Poland; 3 Institute of Organic and Polymer Technology, Department of Chemistry, Wrocław University of Technology Norwida 4/6, 50-373 Wrocław, Poland; 4 Institute of Transplantology, Medical University of Warsaw, ul. Nowogrodzka 59, 02-006 Warsaw, Poland

## Abstract

**Background:**

The antibacterial activity of bacteriophages has been described rather well. However, knowledge about the direct interactions of bacteriophages with mammalian organisms and their other, i.e. non-antibacterial, activities in mammalian systems is quite scarce. It must be emphasised that bacteriophages are natural parasites of bacteria, which in turn are parasites or symbionts of mammals (including humans). Bacteriophages are constantly present in mammalian bodies and the environment in great amounts. On the other hand, the perspective of the possible use of bacteriophage preparations for antibacterial therapies in cancer patients generates a substantial need to investigate the effects of phages on cancer processes.

**Results:**

In these studies the migration of human and mouse melanoma on fibronectin was inhibited by purified T4 and HAP1 bacteriophage preparations. The migration of human melanoma was also inhibited by the HAP1 phage preparation on matrigel. No response of either melanoma cell line to lipopolysaccharide was observed. Therefore the effect of the phage preparations cannot be attributed to lipopolysaccharide. No differences in the effects of T4 and HAP1 on melanoma migration were observed.

**Conclusion:**

We believe that these observations are of importance for any further attempts to use bacteriophage preparations in antibacterial treatment. The risk of antibiotic-resistant hospital infections strongly affects cancer patients and these results suggest the possibility of beneficial phage treatment. We also believe that they will contribute to the general understanding of bacteriophage biology, as bacteriophages, extremely ubiquitous entities, are in permanent contact with human organisms.

## Background

Melanoma and other skin cancers are still among the most serious public health problems. According to the World Health Organization, more than 210,000 skin cancer cases occur every year and about 66,000 patients die as a result. Skin cancers mostly affect humans with light skin; the mortality ratio per 100,000 persons per year is highest in Australia and New Zealand (7.6–7.8), in Europe (1.6–6.4), and in Canada and the United States (3.3–3.8) [1]. This type of cancer is usually characterised with high metastatic activity and relatively high fatality. Besides the constantly emphasised role of early recognition and prevention, surgical removal of tumour and chemotherapy constitute the standard treatment [2]. Surgical procedures and hospital treatment expose cancer patients to a high level of hospital bacterial infections. The risk of hospital bacterial infection is substantial. According to the World Health Organization, between 5% and 10% of patients admitted to hospitals in industrial countries and more than 25% of those in developing countries acquire such infections. This means hundreds of millions of hospital infections every year and a substantial death rate [3]. "Hospital" strains of bacteria are the main representatives of antibiotic-resistant, often multi-drug-resistant, microorganisms. Bacteria are particularly efficient in developing resistance because of their ability to multiply very rapidly and because they can easily transfer their resistance genes (by normal replication and conjugation). Hospitals are a critical component of the antimicrobial resistance problem worldwide. This results from the combination of highly susceptible patients, intensive and prolonged antimicrobial use, and easy cross-infection [4].

Bacteriophages, bacterial viruses unable to infect eukaryotic cells, constitute a serious alternative to antibiotic therapy of bacterial infections [5]. These viruses have been known for almost a hundred years, but renewed interest was noted as the crisis of antibiotic resistance in bacteria became serious. Although phage therapy is limited to only a few therapeutic centres worldwide, the available data documents its high effectiveness and safety. Complete independence from antibiotics' antimicrobial mechanisms was also shown, i.e. bacteriophages do not follow antibiotics' cross-resistance and can be fully effective on antibiotic-resistant bacterial strains [6-9].

The antibacterial activity of bacteriophages has been described rather well and its molecular mechanisms and qualifying agents are also well known. However, knowledge about the direct interactions of bacteriophages with mammalian organisms and their other (i.e. non-antibacterial) activities in mammalian systems is quite scarce. As bacteriophages are unable to infect mammalian cells, they are considered a neutral object characterised by their antigenic properties [10]. It must be emphasised that bacteriophages are natural parasites of bacteria, which in turn are parasites or symbionts of mammals (including humans). This implies a role of mammalian organisms as a special environment for bacteriophages' life cycles. One should expect that bacteriophages adapt to this special "environment" and develop the means of interacting with it. It is known that some phages are more effective in escaping mammalian immune system [11]. These interactions may affect various aspects of immunological and physiological processes and may potentially be advantageous or disadvantageous. Importantly, the possiblebacteriophage circulation in the mammalian body may have a role in the body's defences. Recent findings suggest that bacteriophages may modulate immune functions [12]. These open new perspectives for the understanding of bacteriophage biology and for the development of bacteriophage therapies.

The perspective of the possible use of bacteriophage preparations in cancer patients generates a substantial need to investigate the effects of phages on cancer processes. Interestingly, antimetastatic activity and some inhibition of tumour with T4-like (T4, T2, HAP1) bacteriophage preparations were observed in mice [13,14]. A hypothesis [15] for this unexpected phage activity was proposed with respect to the action of a KGD (Lys-Gly-Asp) amino-acid motif present in gp24 of the T4 phage capsid. KGD is a homologue of the RGD motif which is known to block the activity of beta-3 integrin function in cancer cells. RGD and its homologues are also known disintegrins for alpha(5)beta(1) integrins [16,17]. Both beta-3 integrins, i.e. alpha(v)beta(3) and alpha(IIb)beta(3), and alpha(5)beta(1) mediate cancer cell motility and adhesion and usually promote metastasis and malignancy. They are expressed at high levels in melanoma cells, in contrast to normal melanocytes. Direct engagement in adhesion processes, interactions with extracellular matrix (ECM), and modulation of matrixmetallo-proteinase (MMP) activity in melanoma cells make these integrins among the most important factors mediating melanoma migration [18,19].

Here we report our observations of the effect of T4-like phages on human (Hs294T) and mouse (B16) melanoma migration *in vitro*. The study was intended to provide further necessary data on bacteriophages' activity in cancer processes and to verify previous observations. The *in vivo *anticancer effects of bacteriophages may result from an impact of the investigated preparations on immunological systems (which has to be seriously considered) or from direct interactions with cancer cells. *In vitro *migration excludes the effect of complex mammalian immunology. As T4-like phages are coliphages, their preparations contain lipopolysaccharide (LPS); even highly purified preparations contain a residual amount of LPS [20]. LPS is a potent activator of various processes in mammalian cells. These considerations make studies of the effects of LPS on melanoma migration indispensable. Therefore we investigated its potential effect in all the experiments conducted with bacteriophages, constituting a control for the studies of the bacteriophages themselves.

## Methods

### Bacteriophages

T4 phage was purchased from American Type Culture Collection (ATCC) (Rockville, Maryland, USA). HAP1 (a T4 sub-strain with a high affinity to melanoma cells) was selected at our institute: the Institute of Immunology and Experimental Therapy, Polish Academy of Sciences, Wroclaw (IIET) [13]. The bacteriophages were cultured with *Escherichia coli B *from the Collection of Microorganisms at the IIET. The material comprised highly purified preparations of bacteriophages T4 and HAP1. The bacteriophages were purified by filtration through polysulfone membranes and by two chromatographic techniques: gel filtration on Sepharose 4B (Sigma-Aldrich, Poland) followed by cellulofine sulfate (Millipore, Billerica, USA) chromatography [20]. The purification procedure afforded preparations of phages containing less than 5 U/ml endotoxin for 10^9 ^pfu/ml (lysates: approx. 3000 U/ml), as determined by chromogenic Limulus amebocyte lysate assay (QLC-1000 Chromogenic Endpoint LAL, Bio Whittaker, USA). The phage concentrations were measured by the double-layer method of Adams [21]. The batches prepared by the Bacteriophage Laboratory of the IIET used were: T4_108_, T4_119_, and HAP1_112_, all finally dialysed against phosphate-buffered saline (PBS).

### Lipopolysaccharide (LPS)

LPS was prepared at the IIET. Bacteria were grown for 48 h at 37°C in standard (0.5% NaCl) Luria-Bertani Broth (LB) vigorously aerated by shaking. The bacteria were killed with 0.5% phenol and centrifuged at 39,000 rpm using a flow centrifuge (New Brunswick Scientific, USA) [22]. The bacterial mass was washed three times with distilled water, lyophilised, treated with 90% phenol/water (1:1), and heated to 65°C. LPS was extracted for 15 min according to the method of Westphal and Jann [23]. The extract was cooled to 4°C and centrifuged for 30 min at 3000 × *g*. The water phase was collected. Distilled water was added to the remaining phenol phase and the extraction process was repeated. Both phases were dialysed against water for 72 h (water phase) or for 120 h (phenol phase) and lyophilised. To remove nucleic acids, the resultant LPS was ultra-centrifugated (105,000 × *g*, 6 h, repeated two times), and the LPS suspension was lyophilised again. For the tests, 1 μg/ml of LPS suspension in PBS was prepared by sonication (30 s). The activity of LPS was determined by chromogenic Limulus amebocyte lysate assay (QLC-1000 Chromogenic Endpoint LAL, Bio Whittaker, USA) and it was defined as 4 × 10^4 ^U/ml in the 1-μg/ml preparation. The residual LPS in the bacteriophage preparations allowed a final concentration in the migration assay of 10 U/ml, which equals 0.25 ng/ml. The LPS sample was diluted with PBS to the various desired concentrations (dose gradient); the control for the phage preparations was 10 U/ml.

### Tumour cells

The B16 mouse melanoma cell line and the Hs294T human melanoma cell line were obtained from the ATCC (Rockville, Maryland, USA.). The lines are maintained at the Cell Culture Collection at IIET.

The cells were cultured with normal foetal bovine serum (FBS) media. One day before the migration, the medium was changed: (i) Hs294T was cultured again with medium containing FBS before both fibronectin and matrigel migration and (ii) B16 was cultured in medium containing FBS before the fibronectin migration assay, but in Dulbecco's modified Eagle's medium (DMEM) before the matrigel migration assay as its migration activity was poor and the FBS deficiency stimulated the cells' response to FBS attraction in the migration assay. Before the assay, cells were collected with non-enzymatic Cell Dissociation Solution (Sigma-Aldrich, Poland), centrifuged, resuspended in DMEM (with no FBS), counted in a Burker counting chamber (Roth, Germany) in light microscopy with trypan, and diluted to the desired concentration. The cells were used immediately in the migration assay.

### Migration chamber preparation

Fibronectin assay: 8-μm insert membranes (Falcon BD Biosciences, USA) were sterilely covered with fibronectin (100 μg/ml, Falcon BD Biosciences). Both sides of the membrane were covered with 20 μl of the fibronectin suspension and incubated for 30 min at 37°C. Fibronectin was removed and the inserts were washed three times with sterile water. Subsequently, both sides of the membrane were immersed in a 0.1% albumin solution and incubated for 15 min. The inserts were washed three times with sterile water and dried. The prepared inserts were not stored, but used immediately after preparation.

Matrigel assay: according to the manufacturer's instructions, the 8-μm insert membranes (Falcon BD Biosciences) were covered with matrigel diluted 1:4 with DMEM under sterile conditions, with cooling. Only the upper side of the membrane was covered with 10 μl of the matrigel suspension (i.e. approx. 7 μg/cm^2 ^of the membrane) and slowly dried (overnight in a covered plate) at 37°C. Such prepared inserts can be stored at -20°C. If frozen, they were defrosted at 37°C, and rehydrated with DMEM for 2 hours, and directly applied in the migration assay.

### Migration assay

The cells were suspended in DMEM with no FBS, and applied to the upper section of the migration chamber, with 1 × 10^5 ^Hs294T cells/insert in both the fibronectin and the matrigel assay, 4 × 10^5 ^B16 cells/insert in the matrigel assay, and 5 × 10^5 ^B16 cells/insert in the fibronectin assay. All preparations (bacteriophages, LPS, PBS basic control) were correlated and added at the same final volumes of PBS (125–135 μl), both in the upper and the lower sections of the migration chamber. All the preparations and cells in the upper section were completed with DMEM and with FBS-containing medium to 0.5 ml in the lower section (according to the manufacturer's instructions). Final concentrations of the bacteriophage preparations were 1.5–2.5 × 10^9 ^pfu/ml containing 10 U/ml residual LPS. Concentration of the attracting agent, FBS, in the lower section of the migration chamber was 7.3–7.5%.

The migration was carried out at 37°C with CO_2_. The time of migration was initially optimised and was 2 h for B16 on fibronectin, 7–8 h for B16 on matrigel, 1 h 20 min for Hs294T on fibronectin, and 4.5–5 h for Hs294T on matrigel. After this time (following the manufacturer's instructions) the cells from the upper side of the membrane were removed with a cotton swab. The cells on the bottom side of the membrane were fixed and stained with a Diff-Quick Set (Medion Diagnostics, Düdingen, Switzerland) and counted by light microscopy. The number of cells per membrane was determined, accumulated into groups, and the average was presented.

### Statistical methods

One-way analysis of variance (ANOVA) and the Kruscal-Wallis test with the Statistica 8.0 software package were applied http://www.statsoft.pl.

## Results

### The migration of human and mouse melanoma on fibronectin

Fibronectin is one of the ECM proteins. Its primary function is cell adhesion to the ECM, which is mediated by fibronectin's RGD sequences, and engagement of specific cell surface receptors. It may involve the probable mechanisms of phage action, so the migration studies were initiated with this protein.

**Figure 1:**
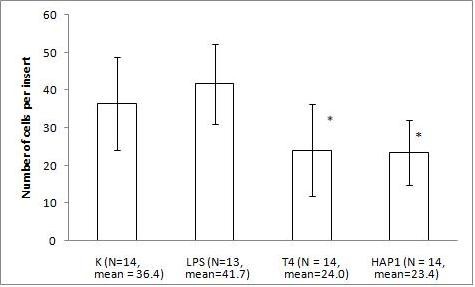
**The effect of T4 and HAP1 bacteriophages on B16 mouse melanoma migration on fibronectin**. The insert: an 8-μm 0.3-cm^2 ^membrane was covered with fibronectin. B16 melanoma cells were applied at 5 × 10^5 ^cells per insert in DMEM. The final concentrations of the bacteriophage preparations were 1.5–2.5 × 10^9 ^pfu/ml and 10 U/ml of residual LPS. The LPS control was also 10 U/ml (which equals 0.25 ng/ml). The concentration of the attracting agent FBS in the lower section of the migration chamber was 7.3–7.5%. Migration was carried out for 2 h at 37°C in CO_2_. The cells were stained and counted under light microscopy on the whole membrane. The mean number of cells per membrane (bars) and SD (lines) are presented.

**Figure 2:**
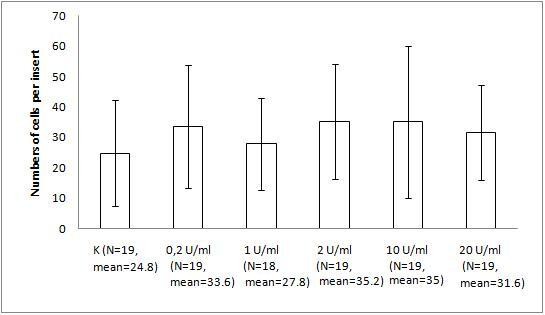
**The effect of LPS on B16 mouse melanoma migration on fibronectin**. The insert: an 8-μm 0.3-cm^2 ^membrane was covered with fibronectin. B16 melanoma cells were applied at 5 × 10^5 ^cells per insert in DMEM. LPS was applied as a dose gradient (10 U/ml, equal to 0.25 ng/ml). The concentration of the attracting agent FBS in the lower section of the migration chamber was 7.3–7.5%. Migration was carried out for 2 h at 37°C in CO_2_. The cells were stained and counted under light microscopy on the whole membrane. The mean number of cells per membrane (bars) and SD (lines) are presented.

The migration assay of B16 melanoma with the bacteriophage preparations and LPS revealed marked and statistically significant inhibition of migration by both T4 phage and HAP1 phage, which was almost the same for both bacteriophages. Migration was inhibited by 34% (p = 0.0235) and 36% (0.0164), respectively, compared with the control and by 42% (p = 0.0008) and 44% (0.0006), respectively, compared with 10 U/ml LPS, identical to the residual LPS content in the phage preparations (Fig. 1). No effect on migration was induced by 10 U/ml LPS (Fig. 1). A gradient of LPS concentrations (0.2–20 U/ml) also did not show any effect on B16 migration activity (Fig. 2).

**Figure 3:**
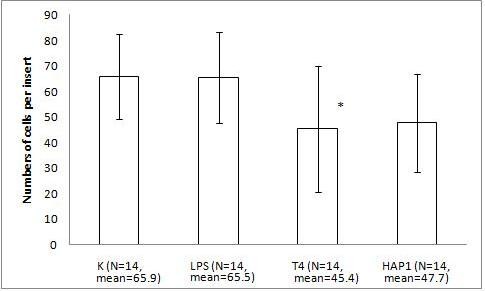
**The effect of T4 and HAP1 bacteriophages on Hs294T human melanoma migration on fibronectin**. The insert: the 8-μm 0.3-cm^2 ^membrane was covered with fibronectin. Hs294T melanoma cells were applied at 1 × 10^5 ^cells per insert in DMEM. The final concentrations of the bacteriophage preparations were 1.5–2.5 × 10^9 ^pfu/ml and 10 U/ml of residual LPS. The LPS control was also 10 U/ml (which equals 0.25 ng/ml). The concentration of the attracting agent, FBS, in the lower section of the migration chamber was 7.3–7.5%. Migration was carried out for 1 h 20 min at 37°C in CO_2_. The cells were stained and counted under light microscopy on the whole membrane. The mean number of cells per membrane (bars) and SD (lines) are presented.

**Figure 4:**
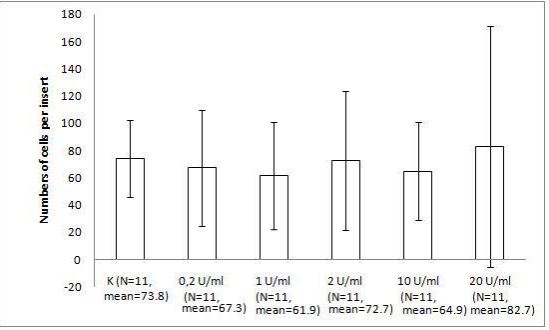
**The effect of LPS on Hs294T human melanoma migration on fibronectin**. The insert: the 8-μm 0.3-cm^2 ^membrane was covered with fibronectin. Hs294T melanoma cells were applied at 1 × 10^5 ^cells per insert in DMEM. LPS was applied as a dose gradient (10 U/ml equals 0.25 ng/ml). The concentration of the attracting agent FBS in the lower section of the migration chamber was 7.3–7.5%. Migration was carried out for 1 h 20 min at 37°C in CO_2_. The cells were stained and counted under light microscopy on the whole membrane. The mean number of cells per membrane (bars) and SD (lines) are presented.

The migration assay of Hs294T melanoma with bacteriophage preparations and LPS revealed response similar to that of the mouse melanoma. Human melanoma was not stimulated by 10 U/ml LPS (the activity was identical to that of the PBS control). Its migration was decreased by 31% (p = 0.0423) by T4 compared with PBS. A significant difference between PBS and HAP1 was not observed (28%, p = 0.0859) (Fig. 3). Expanded analysis of the effect of LPS (dose gradient) showed no significant or marked trend in the human melanoma response (Fig. 4).

### Migration of human and mouse melanoma on matrigel matrix

Matrigel matrix is a reconstituted basement membrane with a wider range of components, including stimulating and regulating factors and various proteins. It allows more complex and multiple interactions of cells during their motility and more complete analysis of the migration process.

**Figure 5:**
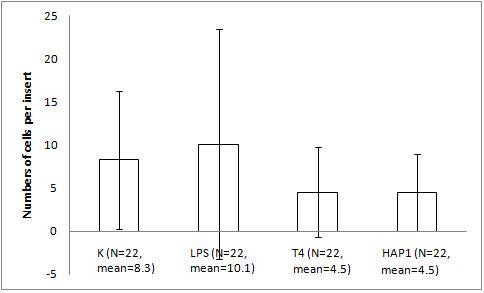
**The effect of T4 and HAP1 bacteriophages on B16 mouse melanoma migration on matrigel matrix**. The insert: the 8-μm 0.3-cm^2 ^membrane was covered with matrigel (approx. 7 μg/cm^2^). B16 melanoma cells were applied at 4 × 10^5 ^cells per insert in DMEM. The final concentrations of the bacteriophage preparations were 1.5–2.5 × 10^9 ^pfu/ml and 10 U/ml of residual LPS. The LPS control was also 10 U/ml (which equals 0.25 ng/ml). The concentration of the attracting agent FBS in the lower section of the migration chamber was 7.3–7.5%. Migration was carried out for 7–8 h at 37°C in CO_2_. The cells were stained and counted under light microscopy on the whole membrane. The mean number of cells per membrane (bars) and SD (lines) are presented.

**Figure 6:**
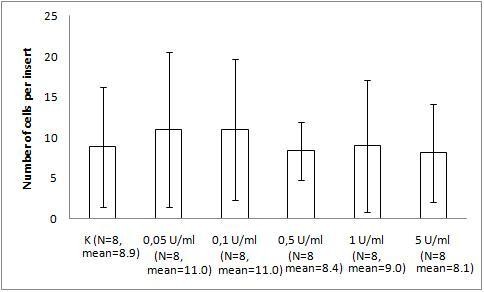
**The effect of low doses of LPS on B16 mouse melanoma migration on matrigel matrix**. The insert: the 8-μm 0.3-cm^2 ^membrane was covered with matrigel (approx. 7 μg/cm^2^). B16 melanoma cells were applied at 4 × 10^5 ^cells per insert in DMEM. LPS was applied as a dose gradient (10 U/ml equals 0.25 ng/ml). The concentration of the attracting agent FBS in the lower section of the migration chamber was 7.3–7.5%. Migration was carried out for 7–8 h at 37°C in CO_2_. The cells were stained and counted under light microscopy on the whole membrane. The mean number of cells per membrane (bars) and SD (lines) are presented.

**Figure 7:**
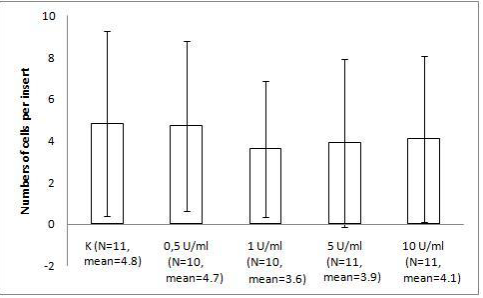
**The effect of LPS on B16 mouse melanoma migration on matrigel matrix**. The insert: the 8-μm 0.3-cm^2 ^membrane was covered with matrigel (approx. 7 μg/cm^2^). B16 melanoma cells were applied at 4 × 10^5 ^cells per insert in DMEM. LPS was applied as a dose gradient (10 U/ml equals 0.25 ng/ml). The concentration of the attracting agent FBS in the lower section of the migration chamber was 7.3–7.5%. Migration was carried out for 7–8 h at 37°C in CO_2_. The cells were stained and counted under light microscopy on the whole membrane. The mean number of cells per membrane (bars) and SD (lines) are presented.

The overall migration activity of B16 melanoma was poor and the results were strongly dispersed. Therefore the assay did not show a significant inhibition of B16 migration by T4 and HAP1 (Fig. 5). The LPS concentration gradient did not reveal any significant trend towards stimulation or inhibition related to the dose series, although the test was made with two complementary sets of doses. The dispersion of the results was also remarkable, which strongly hindered their analysis (Figs. 6 and 7).

**Figure 8:**
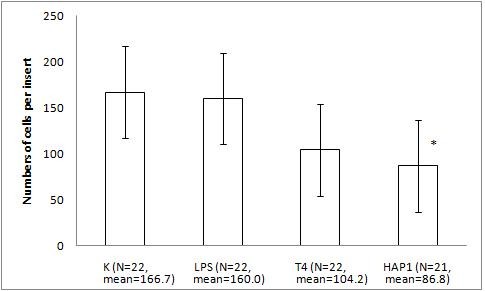
**The effect of T4 and HAP1 bacteriophages on Hs294T human melanoma migration on matrigel matrix**. The insert: the 8-μm 0.3-cm^2 ^membrane was covered with matrigel (approx. 7 μg/cm^2^). Hs294T melanoma cells were applied at 1 × 10^5 ^cells per insert in DMEM. The final concentrations of the bacteriophage preparations were 1.5–2.5 × 10^9 ^pfu/ml and 10 U/ml of residual LPS. The LPS control was also 10 U/ml (which equals 0.25 ng/ml). The concentration of the attracting agent FBS in the lower section of the migration chamber was 7.3–7.5%. Migration was carried out for 4.5–5 h at 37°C in CO_2_. The cells were stained and counted under light microscopy on the whole membrane. The mean number of cells per membrane (bars) and SD (lines) are presented.

**Figure 9:**
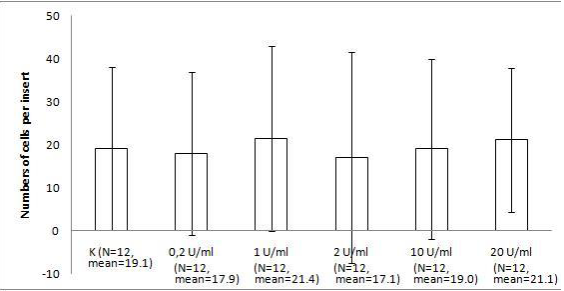
**The effect of LPS on Hs294T human melanoma migration on matrigel matrix**. The insert: the 8-μm 0.3-cm^2 ^membrane was covered with matrigel (approx. 7 μg/cm^2^). Hs294T melanoma cells were applied at 1 × 10^5 ^cells per insert in DMEM. LPS was applied as a dose gradient (10 U/ml equals 0.25 ng/ml). The concentration of the attracting agent FBS in the lower section of the migration chamber was 7.3–7.5%. Migration was carried out for 4.5–5 h at 37°C in CO_2_. The cells were stained and counted under light microscopy on the whole membrane. The mean number of cells per membrane (bars) and SD (lines) are presented.

The migration assay of Hs294T melanoma with the bacteriophage preparations and LPS revealed an inhibition of migration by HAP1 phage by 48% (p = 0.0407). A significant difference between PBS and T4 was not observed (38%, p = 0.0859). Human melanoma migration was not affected by 10 U/ml LPS (Fig. 8). Expanded analysis of the LPS effect (dose gradient) also showed no effect on Hs294T cell response (Fig. 9).

## Discussion

The most important question of this study was the general effect of the bacteriophage preparations on melanoma's migration activity, mostly because of the perspective of developing bacteriophage therapy. The migration of human and mouse melanoma can be inhibited by the purified T4 and HAP1 bacteriophage preparations with no stimulative action, which is plainly an advantageous effect. A response of melanoma cells to LPS (within the investigated range) was not observed and the differences from those of the bacteriophage preparations were marked, so the antimigration activity of the studied preparations cannot be attributed to LPS. It should be pointed out that the LPS content in the purified phage preparation was minimal; in this study the final concentration was 0.25 ng/ml (10 U/ml by the chromogenic Limulus amoebocyte lysate assay).

The high variability of the assay hindered analysis of the observations. The more general assay with matrigel was also much more variable and it ascertained only an inhibitory effect of HAP1 on Hs294T migration. In the fibronectin assay, significant inhibition was observed both for the mouse (T4 and HAP1) and human (T4) melanoma. This is in line with the hypothesis on the RGD-engaging mechanism of changes in cell migration [15] as cell adhesion to the ECM is mediated by fibronectin's RGD sequences. Integrins alpha(v)beta(3), alpha(IIb)beta(3), and alpha(5)beta(1) mediate cancer cell motility and adhesion and are susceptible to the activity of RGD homologues. They are known to promote metastasis and malignancy and to be highly expressed in melanoma cells (in contrast to normal melanocytes). Alpha(v)beta(3) and beta(1)-integrins are highly expressed at the leading edge of invasive explants. They also regulate MMPs functions that are critical for the invasive properties of tumour cells as they degrade ECM components [18,19]. The overall mechanism of melanoma motility is obviously complex and engages a wider range of surface particles. Other factors strongly associated with melanoma development and progression that also play roles in melanoma adhesion and motility are melanoma cell adhesion molecule (Mel-CAM, MUC18, CD146), L1 cell adhesion molecule (L1-CAM, CD171), activated leukocyte cell adhesion molecule (ALCAM, CD166), vascular cell adhesion molecule 1 (VCAM-1, CD106), intracellular cell adhesion molecule 1 (ICAM-1, CD54), and carcinoembryonic antigen-related cell adhesion molecule 1 (CEACAM1, CD66a) [19]. One cannot preclude unknown functions of the above factors in the inhibitory action of T4-like phage preparations on melanoma migration. Nevertheless, current knowledge (both laboratory observations and theoretical analyses) does not justify any assumptions regarding their interaction with bacteriophages. Some of the above surface particles interact via beta(3)-integrin subunits; for example, L1-CAM mediates melanoma cell/melanoma cell and melanoma cell/endothelial cell interactions [24]. Therefore, L1-CAM can be indirectly engaged in the studied effect. We consider the problem of molecular mechanisms of phage-melanoma interaction still open and believe that further investigations are needed.

Models of *in vitro *studies allow investigating the direct effects of preparations on migrating cells. This brings us closer to understanding previously observed *in vivo *antimetastatic effects [13,14]. The *in vivo *anticancer effects may result from an impact of the investigated preparations on immunological systems, which has to be seriously considered. *In vitro *migration excludes the effect of complex mammalian immunology. Observations of the "antimigratory" effect of bacteriophages suggest that they are able to influence (at least some) cancer cells directly. Previously we investigated the interactions of bacteriophage T4 with mammalian cells, observing an unexpected ability of the bacteriophage to bind weakly to melanoma cells *in vitro*. We selected bacteriophage HAP1, which was able to bind cancer cells more strongly. Importantly, HAP1 was also much more effective against melanoma metastases *in vivo *[13]. A mutation in the *hoc *gene that differentiates bacteriophage HAP1 and its parental strain T4 was found [14]. Nevertheless, in these studies we did not find any difference in the effects of T4 and HAP1 on melanoma migration *in vitro*. This may suggest that some immunological components are engaged in the activity of HAP1. This phage is different (from T4 phage) in, among other properties, the time and means of clearance from a mammalian organism, which may contribute to these observations. On the other hand, the difference between T4 and HAP1 interactions with melanomas may simply be undetectable in the types of tests conducted.

We believe that our observations are of importance for any further attempts to use bacteriophage preparations in antibacterial treatment. To the best of our knowledge, there are no published data on the effect of bacteriophages on macrophage or lymphocyte (normal cell) migration *in vitro*. We also work on this issue and we hope to be able to present data in the future. It should be pointed out that bacteriophages constitute a strongly diversified group of microorganisms and our observations apply to T4-like phages. Other types of bacteriophages (with different genetics and protein construction) must be investigated and analysed independently. As the risk of antibiotic-resistant hospital infections strongly affects cancer patients, we consider that such investigations are greatly needed. We also believe that they will contribute to the general understanding of bacteriophage biology. Bacteriophages, extremely ubiquitous entities, are in permanent contact with human organisms. They are present in water, food, and soil and constitute a part of the "microbial flora" of human skin and gastrointestinal tract and penetrate all tissues. Knowledge about their influence on the human body may be very useful, similar to the knowledge about the "beneficial" strains of bacteria that make up our microflora. There may also be some "beneficial" bacteriophages in our bodies and in our environment.

## Conclusion

The migration of human and mouse melanoma can be inhibited by purified T4 and HAP1 bacteriophage preparations. A response of melanoma cells to LPS (within the investigated range) was not observed, so the antimigration activity of the studied preparations cannot be attributed to LPS. No differences in the effects of T4 and HAP1 on melanoma migration were observed.

## Authors' contributions

KD: design and planning of the experiments, migration assays, cell cultures and preparation, bacteriophage sample preparation, LPS sample preparation, results analysis, drafting the manuscript; GS: migration assays, results analysis; PJ: migration assays, cell cultures and preparation; AK: migration assays, JW: cell culture design and control, consultation on cancer cell lines, media, BO: purification of bacteriophages, LPS content determination by chromogenic Limulus amoebocyte lysate assay, MZ: purification of bacteriophages, LPS content determination by chromogenic Limulus amoebocyte lysate assay, KSJ: LPS purification, JB: bacteriophage purification process, GP: preparation of the membranes used in bacteriophage purification, MM: cell cultures and media, AG: design and planning of the experiments, results analysis, manuscript revision.
